# Hydroxylation of *N*-acetylneuraminic Acid Influences the *in vivo* Tropism of *N*-linked Sialic Acid-Binding Adeno-Associated Viruses AAV1, AAV5, and AAV6

**DOI:** 10.3389/fmed.2021.732095

**Published:** 2021-12-21

**Authors:** Estrella Lopez-Gordo, Alejandro Orlowski, Arthur Wang, Alan Weinberg, Susmita Sahoo, Thomas Weber

**Affiliations:** ^1^Cardiovascular Research Institute, Icahn School of Medicine at Mount Sinai, New York City, NY, United States; ^2^Department of Population Health Science and Policy, Icahn School of Medicine at Mount Sinai, New York City, NY, United States; ^3^Graduate School of Biomedical Sciences, Icahn School of Medicine at Mount Sinai, New York City, NY, United States

**Keywords:** adeno-associated virus, N-acetylneuraminic acid, N-glycolylneuraminic acid, sialic acid, tropism, animal model

## Abstract

Adeno-associated virus (AAV) vectors are promising candidates for gene therapy. However, a number of recent preclinical large animal studies failed to translate into the clinic. This illustrates the formidable challenge of choosing the animal models that promise the best chance of a successful translation into the clinic. Several of the most common AAV serotypes use sialic acid (SIA) as their primary receptor. However, in contrast to most mammals, humans lack the enzyme CMAH, which hydroxylates cytidine monophosphate-*N*-acetylneuraminic acid (CMP-Neu5Ac) into cytidine monophosphate-*N-*glycolylneuraminic acid (CMP-Neu5Gc). As a result, human glycans only contain Neu5Ac and not Neu5Gc. Here, we investigate the tropism of AAV1, 5, 6 and 9 in wild-type C57BL/6J (WT) and CMAH knock-out (CMAH^−/−^) mice. All *N*-linked SIA-binding serotypes (AAV1, 5 and 6) showed significantly lower transduction of the heart in CMAH^−/−^ when compared to WT mice (5–5.8-fold) and, strikingly, skeletal muscle transduction by AAV5 was almost 30-fold higher in CMAH^−/−^ compared to WT mice. Importantly, the AAV tropism or distribution of expression among different organs was also affected. For AAV1, AAV5 and AAV6, expression in the heart compared to the liver was 4.6–8-fold higher in WT than in CMAH^−/−^ mice, and for AAV5 the expression in the heart compared to the skeletal muscle was 57.3-fold higher in WT than in CMAH^−/−^ mice. These data thus strongly suggest that the relative abundance of Neu5Ac and Neu5Gc plays a role in AAV tropism, and that results obtained in commonly used animal models might not translate into the clinic.

## Introduction

Adeno-associated viruses (AAVs) are non-pathogenic, have relatively low immunogenicity, and trigger long-term transgene expression in non-dividing cells, even in the absence of integration of the viral DNA into the host genome. These features make them lead candidates for gene therapy and, over the last few years, AAV-based vectors have found their way into the clinic. In addition to a number of oligonucleotide-based therapies, only two *in vivo* gene therapy treatments are currently approved by the FDA, and both are based on AAV: (1) Luxturna, for the treatment of the early childhood blindness disease Leber's congenital amaurosis type 2 and (2) Zolgensma for the treatment of spinal muscular atrophy, a debilitating disease where afflicted children usually die or require mechanical ventilation before age 2.

For AAV gene therapy, transduction of the correct cell type is essential. Unfortunately, the use of isolated primary cells as a screening platform to determine *in vivo* tropism of AAV is not a promising approach. For instance, upon tail vein injection, AAV9 transduces rat cardiomyocytes 10-fold more efficiently than AAV6. In contrast, AAV6 transduction of freshly isolated adult rat cardiomyocytes is at least 100-fold higher than that of AAV9 (Rapti K. and Weber T., unpublished results).

Furthermore, assessment of AAV tropism *in vivo* can also show dramatic differences among species (1), and even within the same species AAV tropism can vary among strains. For instance, brain transduction upon systemic injection of the AAV9 variant AAV-Ph.B differed <2-fold among C57BL/6N, SIL/J, FVB/N, and DBA/2 mice when compared to C57BL/6J (2). Astonishingly, however, transduction of the brain of BALB/c mice was virtually non-existent (2). These differences in AAV tropism are caused, at least in part, by species-dependent differences in tissue distribution of AAV receptors, differences in intracellular trafficking of AAV particles, vector stability within the cell and efficiency of escape from the vasculature into the surrounding tissue/organ (2–5). Therefore, it might not be surprising that a significant amount of successful preclinical studies did not yield the therapeutic efficacy observed in small and large animal studies. A good example is the Calcium Upregulation by Percutaneous administration of gene therapy In cardiac Disease (CUPID) trial (6). Whereas the delivery of AAV1 encoding the sarcoplasmic calcium ATPase, SERCA2a, resulted in a significant improvement of cardiac function in rat and pig models of heart failure (7, 8), it did not significantly improve the clinical outcome in patients with heart failure in phase 2b of the CUPID trial (6). As shown by the authors, the failure in therapeutic efficacy was likely due to the inefficient transduction of human cardiomyocytes, since an approximate conversion of number of vector genomes (vg)/μg of total DNA to vg/diploid host genome suggests that <1% of cardiomyocytes contained a vector genome (6). These studies highlight the importance of performing preclinical studies in animal models that both mimic the clinical features of the human disease as well as the cellular tropism of the AAV serotype/variant in humans. Moreover, the tragic death of three children in a clinical trial for the treatment of X-linked myotubular myopathy (XLMTM) with an AAV8 vector delivering a functional copy of *MTM1* (NCT03199469) is a sobering reminder that the injection of ever higher vector doses is not a solution for the poor transduction efficiencies of currently available AAVs. Instead, it highlights the need for the isolation/design of AAVs with improved vector performance. To achieve this, it is paramount that we have animal models that faithfully mirror the tropism and transduction efficiencies of AAV serotypes and variants.

Since the first AAV was isolated from adenovirus preparations in 1965 (9), multiple serotypes (AAV1–AAV13) have been identified and, for most of the serotypes, the primary receptors they use *in vitro* have been described. Among AAV1–AAV13, heparan sulfate proteoglycans (HSPGs) and sialic acids (SIAs) are by far the most commonly used receptors. For instance, AAV2, 3, 6, and 13 were shown to use HSPGs, whereas AAV1, 4, 5, and 6 use SIA, and AAV9 uses β1–4 *N*-linked galactose (10, 11). Interestingly, although AAV1, AAV5, and AAV6 share the same receptor (sialic acid), they bind to it in a different manner due to differences in their capsid surface topology (5). For instance, AAV5 binds to α2–3 *N*-linked SIA (11–13), and AAV1 and 6 to α2–3 and α2–6 *N*-linked SIA (11, 14). In addition, several co-receptors have been described as being necessary for AAV entry into host cells following attachment to the primary receptor (15), although the importance of many co-receptors for AAV transduction remains controversial. For instance, none of the proposed AAV2 co-receptors showed up as being essential for AAV2 transduction in a haploid knockout screen (16). On the other hand, in the same screen, KIAA0319L was identified as an essential host factor for AAV2 transduction. Since KIAA0319L binds to AAV at the plasma membrane, it is commonly referred to as AAV receptor (AAVR) (16), although AAVR is not required for AAV endocytosis (17). Significantly, multiple serotypes including AAV1, 2, 3b, 5, 6, 8, and 9 are dependent on AAVR for both *in vitro* and *in vivo* transduction (16–19).

**Figure 1:**
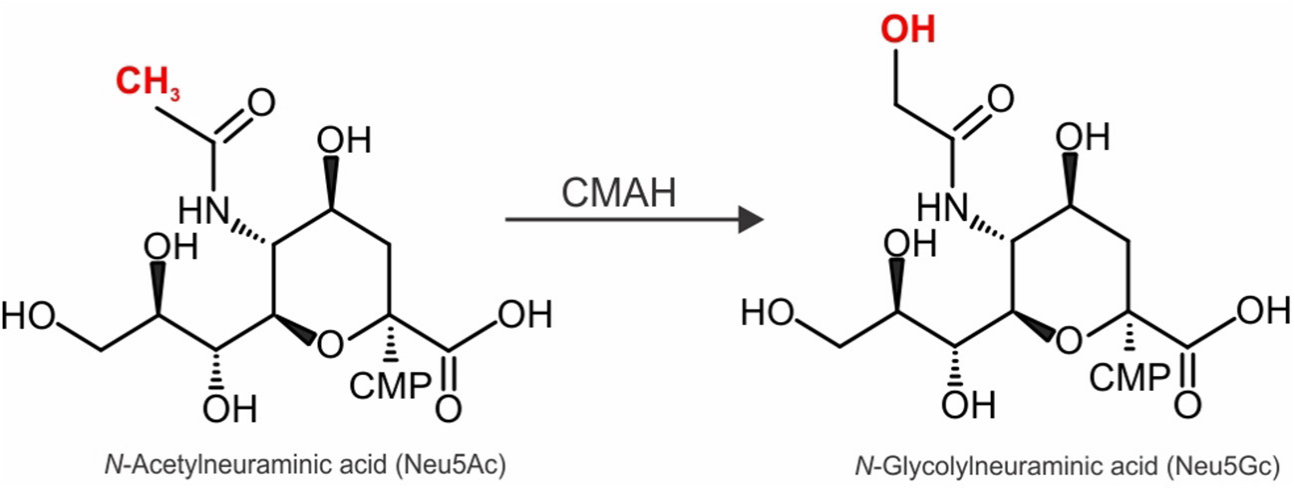
Enzymatic activity of CMAH and its effect on complex *N*-linked glycan structure. Schematic representation of the conversion of sialic acid cytidine monophospho-*N*-acetylneuraminic acid (CMP-Neu5Ac) to cytidine monophospho-*N*-glycolylneuraminic acid (CMP-Neu5Gc) catalyzed by the CMP-Neu5Ac hydroxylase (CMAH).

Due to the differences in AAV receptor usage, it becomes essential to consider the species-specific receptor abundance and expression patterns when choosing an animal model that is most likely to mimic the AAV tropism in humans (3, 4, 20). Interestingly, the 1% of protein sequences that differ between humans and chimpanzees includes several proteins related to SIA biology (20, 21). One of the most intriguing differences is the absence of a functional cytidine monophospho-*N*-acetylneuraminic acid hydroxylase (CMAH) in humans (22, 23). CMAH hydroxylates cytidine monophosphate-*N*-acetylneuraminic acid (CMP-Neu5Ac) to generate cytidine monophosphate-*N-*glycolylneuraminic acid (CMP-Neu5Gc) (Figure 1). Neu5Ac and Neu5Gc are among the most prevalent forms of SIA found in most mammals (24–26). Interestingly, humans lost CMAH around 3 million years ago following human divergence from the chimpanzee lineage (27–29) due to a 92 bp deletion that caused a frameshift and thus premature termination of the polypeptide chain (22, 23). Thus, while gorillas, orangutans, chimpanzees and bonobos display Neu5Gc on their cells, human cells lack Neu5Gc (27–29). Stunningly, New World monkeys (e.g., marmosets) also lost CMAH expression after their phylogenetic separation from a common ancestor of Old World Monkeys and Humans (30). Thus, it is possible that the loss of CMAH conferred our early ancestors with resistance to infection by certain pathogens, since many pathogens rely on SIA as their receptors (21, 31). Other mammals that lost CMAH expression include ferrets and guinea pigs (30–33). Interestingly, marmosets, ferrets and guinea pigs can transmit Influenza A (34–36), which uses SIA as a receptor (37). Given these data, and the fact that AAV1 also uses SIA as a receptor, it seems reasonable to ask whether the absence of CMAH played a role in the failure of clinical trials such as the CUPID trial.

Currently, rhesus macaques appear to be the most commonly used animal species for toxicology, organ-specific transgene expression and biodistribution studies with AAVs before proceeding to clinical trials. However, as pointed out above, all Old World monkeys, including rhesus macaques, express functional CMAH. Since the use of animal models that mimic the tropism of AAV serotypes and variants in humans are key for the development of AAV gene therapy, we here test the hypothesis that AAV1, AAV5 and AAV6, all of which use SIA as their primary receptor, display different tropism in CMAH knockout mice when compared to wild-type C57BL/6J (WT) mice. AAV9, which uses galactose as its primary receptor, served as a control. Our study sheds new light onto the selection of appropriate animal models for the assessment of AAV vectors for gene therapy.

## Materials and Methods

### Preparation of Recombinant Vectors

Recombinant AAV (rAAV)1-Luc, rAAV5-Luc, rAAV6-Luc, and rAAV9-Luc were produced as previously described (38). The only differences are that the day after transfection, the medium was replaced with fresh DMEM medium containing 2% FBS, and the 17% iodixanol solution was replaced by a 15% solution. pDG1, pDG5, pDG6, and pDG9 were used to produce rAAV1-Luc, rAAV5-Luc, rAAV6-Luc, and rAAV9-Luc, respectively.

### Animal Procedures

All animal experiments were approved by the Institutional Animal Care and Use Committee (IACUC) of Icahn School at Mount Sinai and adhered with the Guide for the Care and Use of Laboratory Animals from the NIH. Animals were housed in the facility under controlled environmental conditions where temperature was maintained at ambient temperature with 12 h/12 h light/dark cycles. Mice were provided with nesting material, fed standard chow, and water was provided *ad libitum*.

For *in vivo* experiments, adult 8-weeks-old male and female wild-type C57BL/6J (WT) and CMAH knockout (CMAH^−/−^) mice (39) were used. WT C57BL/6J (cat. no.: 000664) and a CMAH^−/−^ breeding pair [B6.129X1-Cmah^tm1Avrk^/J (cat. no.: 017588)] were purchased from The Jackson Laboratory. A total of 60 μl of rAAV-Luc (5e9 vg/μl in lactated Ringer solution) were injected into the tail vein. Mice were sacrificed 1 month following AAV administration using CO_2_, and the heart, liver, quadriceps skeletal muscle, brain, kidney, and lung were immediately harvested and stored at −80°C. WT mice: AAV1, 6 and 9 (*n* = 5 females and 5 males), AAV5 (*n* = 5 females and 5 males for heart, skeletal muscle, brain, kidney, and lung; *n* = 3 females and 5 males for liver). CMAH^−/−^ mice: AAV1 (*n* = 5 females and 5 males for heart, skeletal muscle, brain, kidney, and lung; *n* = 4 females and 5 males for liver), AAV5 (*n* = 5 females and 5 males for liver, skeletal muscle, brain, kidney, and lung; *n* = 4 females and 5 males for heart), AAV6 and 9 (*n* = 5 females and 5 males).

### Luciferase Assay

Luciferase activity was quantified for each organ as previously described (38). The only difference is that approximately 25 mg of tissue samples were each resuspended in 500 μl of lysis buffer (25 mM Tris-hydrochloride pH 8, 2 mM DTT, 2 mM EDTA, 10% glycerol, 1% Triton X-100).

### Western Blotting Assays

The abundance of SIA-containing glycoproteins and their staining profile was assessed by lectin blotting of heart, liver, skeletal muscle, brain, kidney and lung protein extracts of WT or CMAH^−/−^ male mice. Tissues were crushed as previously described (40), and ~25 mg were resuspended in 1 ml of RIPA lysis buffer (cat. no.: 89900; Thermo Fisher Scientific) containing Complete Mini EDTA-free Protease Inhibitor Cocktail (cat. no.: 11836170001; Sigma-Aldrich) to lyse the tissue. The mix was vortexed for 15 s, agitated at room temperature (r.t.) for 15 min and freeze-thawed three times from −80 to 37°C with brief vortexing after each thaw cycle. Protein lysates were sonicated 3 times with 3 pulses of 20 kHz with a 1 min rest on ice between each sonication, and spun at 12,000 x *g* for 3 min to pellet debris. The supernatant was stored at −80°C and the protein concentration was determined by a BCA protein assay (cat. no.: 23227; Pierce by Thermo Fisher Scientific). Protein extracts [15 μg (heart), 20 μg (liver, skeletal muscle and brain) or 8 μg (kidney and lung)] of WT or CMAH^−/−^ mice were mixed with NuPAGE LDS Sample buffer (cat. no.: NP0007; Invitrogen by Thermo Fisher Scientific) and 100 mM DTT, heated for 10 min at 70°C and loaded onto a 10% SDS-PAGE gel. Protein bands were transferred using a wet-blotting system onto a 0.2 μm Amersham Protran nitrocellulose Western blotting membrane (cat. no.: 45-004-011; Cytiva by Fisher Scientific) at 25 V and 4°C overnight. The membrane was washed with PBS for 5 min, blocked with 2% BSA PBS for 60 min at r.t., washed 3 times with PBS-Tween for 5 min each, and incubated with Maackia Amurensis Lectin (MAL) II lectin (cat. no.: B-1265; Vector Laboratories) that binds to α2-3 linked SIA (20 μg/ml) or Sambucus Nigra Lectin (SNA) (cat. no.: B-1305-2; Vector Laboratories) that binds to α2-6 linked SIA and to a lesser extent α2–3 linked SIA (10 μg/ml) in PBS for 60 min at r.t. Next, the membrane was washed as before, incubated with HRP-streptavidin (cat. no.: 405210; BioLegend) in a 1:2,000 dilution for 60 min at r.t., washed again, and developed using the SuperSignal West Pico Plus Chemiluminescent Substrate kit (cat. no.: 34580; Thermo Scientific by Fisher Scientific).

### Immunohistochemistry Assays

Heart, liver, skeletal muscle, brain, kidney and lung of WT or CMAH^−/−^ male and female mice were fixed with 4% PFA overnight at 4°C. Fixed organs were paraffin-embedded, and 6 μm tissue sections were dried overnight at 37°C and incubated at 55°C for 45 min. Tissue sections were de-paraffinized and rehydrated through xylenes and ethanol, washed 3 times with PBS-Tween for 3 min each, and antigens retrieved with Tris-based Antigen Unmasking Solution (cat. no.: H-3301-250) by boiling sections for 5 min in a pressure cooker. After sections cooled down to r.t., endogenous biotin and streptavidin were blocked with Streptavidin/Biotin blocking kit (cat. no.: SP-2002), sections were washed as before, and non-specific binding was blocked with Carbo-Free Blocking solution (cat. no.: SP-5040) for 30 min at r.t. For lectin staining, sections were incubated with MAL II to bind α2–3 linked SIA or with SNA, which binds α2-6 linked SIA and to a lesser extent α2–3 linked SIA, at 10 μg/ml in PBS for 30 min at r.t. For Neu5Gc staining, sections were incubated with chicken anti-Neu5Gc primary antibody (cat. no.: 146903, BioLegend) or chicken IgY isotype control (cat. no.: 402101, BioLegend) at 1 μg/ml in Carbo-Free Blocking solution at 4°C overnight. On the following day, sections were washed 3 times as before and incubated with biotinylated goat anti-chicken IgY secondary antibody (cat. no.: BA-9010, Vector Laboratories) at 7.5 μg/ml in PBS for 60 min at r.t. Next, for both lectin and Neu5Gc staining, sections were washed as before, incubated with VECTASTAIN ABC-AP reagent (cat. no.: AK-5000), washed in PBS-Tween for 5 min, and incubated with Vector Blue Alkaline phosphatase Substrate (cat. no.: SK-5300) in the dark for 25 min (when MAL II or antibodies were used) or 15 min (when SNA was used). Sections were washed in PBS for 5 min, rinsed with tap water, nuclei counterstained for 5 min with VECTOR Nuclear Fast Red (cat. no.: H-3403), and rinsed again for 10 min. Sections were dehydrated and clarified through ethanol and Histo-Clear II (cat. no.: HS2021GLL; National Diagnostics), and slides were mounted using VectaMount Permanent Mounting medium (cat. no.: H-5000). Images were acquired with a Leica DMi8 imaging system using Application Suite X software and a DCM2900 camera. For lectin staining, male and female WT and CMAH^−/−^ mice sections were stained simultaneously for each organ. For Neu5Gc staining, all sections were stained simultaneously. Adjustments were applied equally to all acquired images. Unless otherwise stated, all reagents used were purchased from Vector Laboratories.

### Statistical Analysis

**Figure 2:**
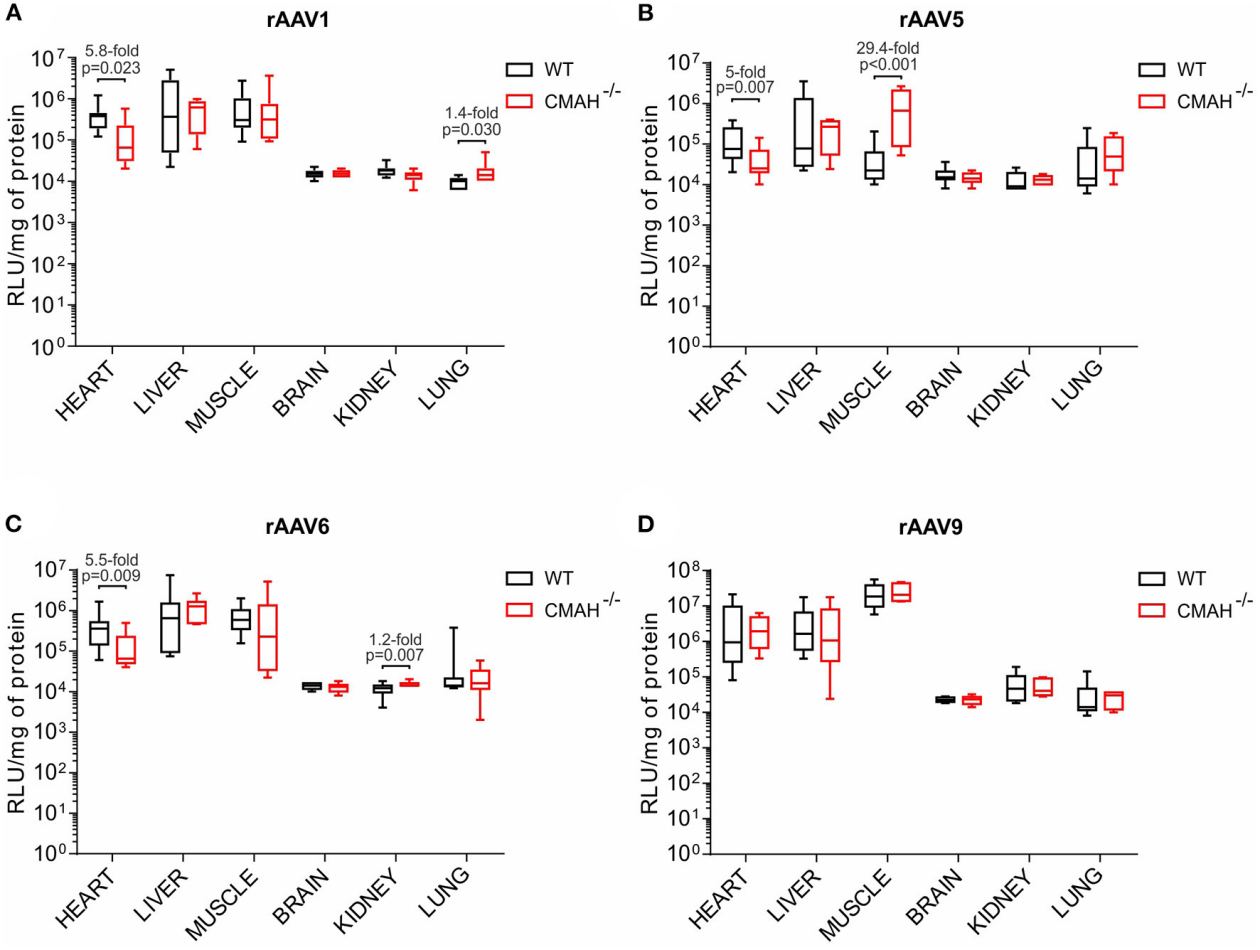
Transduction efficiencies of AAV1, AAV5, AAV6, and AAV9 in wild-type C57BL/6J (WT) and CMAH knockout (CMAH^−/−^) mice. WT (*n* = 8-10) or CMAH^−/−^ (*n* = 9-10) mice were tail vein injected with 3 × 10^11^ vg of rAAV1-Luc **(A)**, rAAV5-Luc **(B)**, rAAV6-Luc **(C)**, or rAAV9-Luc **(D)**. One month post vector administration, luciferase activity and protein content were determined in each organ as described in the Materials and Methods section. Luciferase activity values were normalized to total protein content and expressed as relative light units (RLU)/mg of protein. Boxes correspond to upper and lower quartiles, the horizontal line represents the median, and the whiskers mark the minimum and maximum values. *P*-values among the groups are indicated. The Mann-Whitney test was applied for statistical analysis.

**Figure 3:**
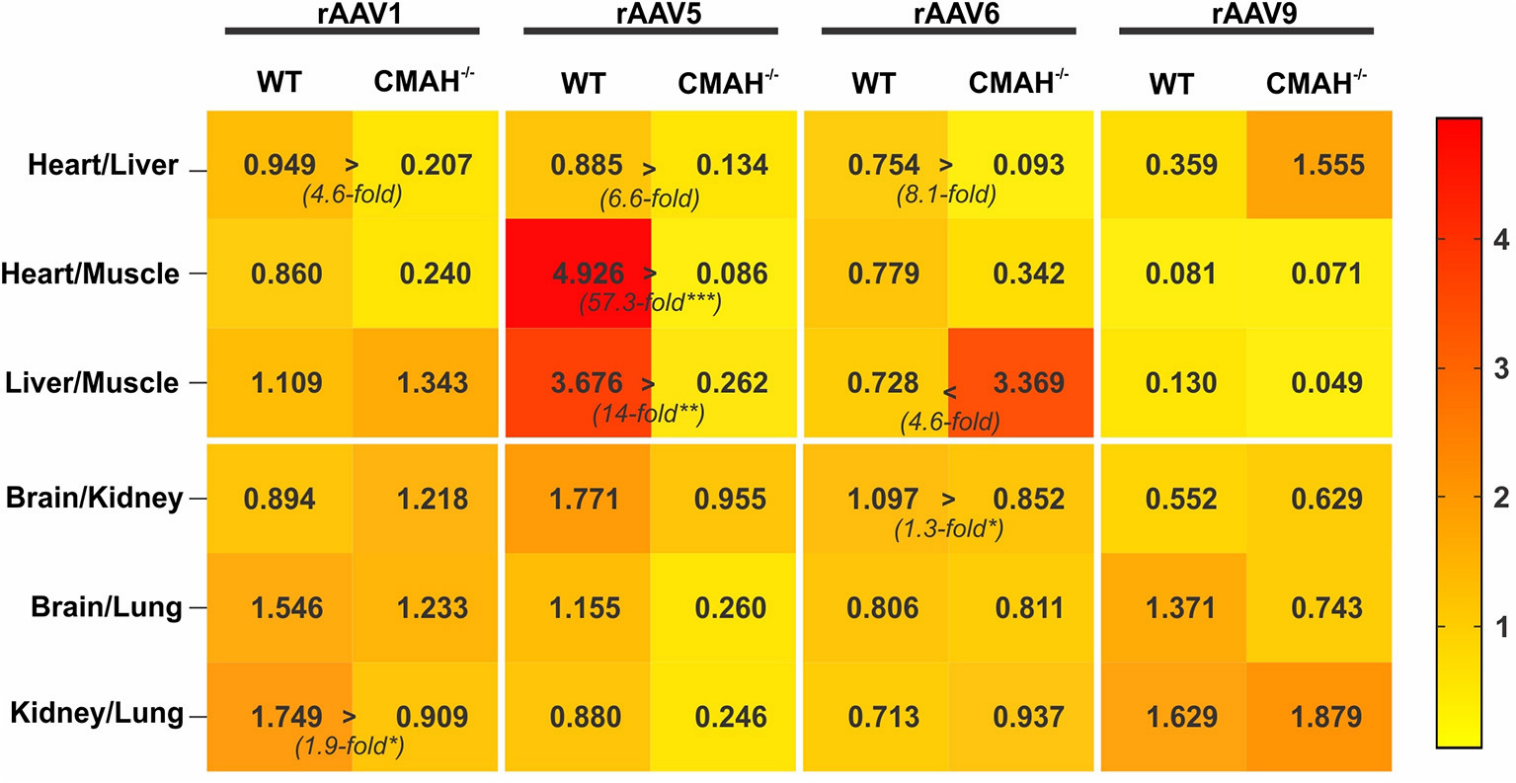
Tropism of AAV1, AAV5, AAV6, and AAV9 in wild-type C57BL/6J (WT) vs. CMAH knockout (CMAH^−/−^) mice. WT (*n* = 8-10) or CMAH^−/−^ mice (*n* = 9-10) were tail vein injected with 3 × 10^11^ vg of rAAV1-Luc, rAAV5-Luc, rAAV6-Luc, or rAAV9-Luc. The relative transduction of organ pairs in mice (WT or CMAH^−/−^) is shown as a heatmap. The numbers in the squares correspond to the median of the ratio of relative luminescence normalized to mg of protein between the organ pairs, as indicated. ^*^p < 0.05, ^**^p < 0.01, ^***^p < 0.001.

For Figure 2, the Shapiro-Wilk test demonstrated that luciferase expression data did not present a normal distribution. Thus, the Mann-Whitney test was performed for significance assessment using version 7 of Prism for Mac (Graphpad). *p* < 0.05 was considered statistically significant. For Figure 3, the level of luciferase expression in major mice organs were compared via ratio. The ratios were ranked and mouse strains were compared via the non-parametric Wilcoxon rank sum test. For statistical analysis, data was analyzed using the SAS System software 9.4 (SAS Institute, Inc., Cary, N.C.).

## Results

### The Lack of CMAH Does Not Affect the Tissue Profile of α2–3 and α2–6 *N*-Linked SIA

First, we confirmed that, in contrast to wild-type C57BL/6J (WT) mice, CMAH knockout (CMAH^−/−^) mice lack Neu5Gc, as previously reported (39, 41), by performing immunostaining of Neu5Gc in the major organs (heart, liver, skeletal muscle, brain, kidney, and lung). As expected, WT female and male mice showed positive staining for Neu5Gc compared to the isotype control for all the tissues assessed (Supplementary Figure 1). In contrast, male CMAH^−/−^ mice showed levels of staining comparable to the isotype control (Supplementary Figure 1A). Overall, female CMAH^−/−^ mice showed a pattern of staining comparable to that observed for male CMAH^−/−^ mice except for skeletal muscle, kidney, and lung tissue, where the staining with the anti-Neu5Gc and isotype control antibodies showed differences (Supplementary Figure 1B).

Next, to confirm that the lack of CMAH in CMAH^−/−^ mice only results in the absence of Neu5Gc (39) and does not affect α2–3 *N*-linked SIA [receptor for AAV1, 5 and 6 (11, 13)] or α2-6 *N*-linked SIA [receptor for AAV1 and 6 (11)], we analyzed the expression pattern and abundance of α2–3 and α2–6 *N*-linked SIA in the major organs in WT and CMAH^−/−^ mice. As can be seen from Supplementary Figure 2, lectin blotting of tissue lysates showed, at most, subtle differences in the abundance of α2–3 linked SIA and α2–6 linked SIA in the organs tested. Similarly, when we performed histology to assess the tissue distribution profile of SIA-containing glycoproteins, we observed no dramatic differences in the staining pattern of α2–3 or α2–6 linked SIA between WT and CMAH^−/−^ male and female mice for any of the tissues assessed (Supplementary Figure 3). These results confirmed that the expression pattern and abundance of α2-3 and α2-6 *N*-linked SIA in the major organs is comparable between WT and CMAH^−/−^ mice.

### Differential Tropism of *N*-Linked SIA-Binding AAV1, 5, and 6 in CMAH^–/–^ Mice

To assess AAV tropism in an animal model that has a similar SIA composition than that in humans, we injected AAV1, 5, 6, and 9 intravenously into WT or CMAH^−/−^ mice. CMAH^−/−^ mice lack the enzyme responsible for converting Neu5Ac into Neu5Gc by adding a hydroxyl group onto the activated sugar nucleotide form of Neu5Ac (CMP-Neu5Ac) (Figure 1). First, localization and intensity of luciferase expression was assessed by *in vivo* imaging of luciferase activity in male WT and CMAH^−/−^ mice (data now shown). All animals showed bioluminescence from the upper abdominal regions on the ventral axis. AAV9 showed a more general distribution throughout the body, including the hindlimb region, head and thoracic cavity. Unfortunately, due to very high expression of luciferase in the liver, expression from other organs such as the heart or lungs was not distinguishable from expression in the liver. Based on these results, we sacrificed the mice at day 30 and analyzed luciferase expression in the major organs from tissue lysates.

In the heart, AAV1, 5 and 6 showed 5.8, 5, and 5.5-fold lower transduction levels in CMAH^−/−^ mice compared to WT mice, respectively (Figures 2A–C). Interestingly, there were no statistically significant differences in the transduction levels in liver between WT and CMAH^−/−^ mice for any of the AAV serotypes tested (Figures 2A–C). In skeletal muscle, AAV1 and AAV6 transduced WT and CMAH^−/−^ mice with comparable efficiency. The most dramatic difference in transduction that we observed was for AAV5, where transduction of skeletal muscle was 29.4-fold higher (*p* < 0.001) in CMAH^−/−^ mice compared to WT mice (Figure 2B). Expression in the brain, kidney and lung was overall much lower than that observed in heart, liver and skeletal muscle for all the serotypes tested (Figures 2A–D). In these organs, the only statistically significant difference observed in transduction levels between WT and CMAH^−/−^ mice was for AAV1 in lung (1.4-fold higher in CMAH^−/−^ mice; Figure 2A) and for AAV6 in kidney (1.2-fold higher in CMAH^−/−^ mice; Figure 2C).

Since AAV9 uses β1–4 *N*-linked galactose as a receptor (10), this serotype was used as a control. As expected, no statistically significant differences in transduction were observed for AAV9 (Figure 2D), since the lack of expression of CMAH in mice does not affect the abundance of other sugars such as galactose.

Tropism of an AAV serotype is defined by specific variations in transduction efficiency of different organs within a given species/strain. To visualize the differences in tropism between WT and CMAH^−/−^ mice among AAV serotypes we created a heat map showing the transduction ratios between organ pairs (Figure 3). The differences in tropism for AAV1, 5 and 6 among the organs expressing high levels of luciferase (heart, liver and skeletal muscle) were overall more pronounced than those for organs expressing lower levels of luciferase (Figure 3). For instance, the heart:liver transduction ratios for AAV1, 5 and 6 were 4.6, 6.6, and 8.1-fold higher in WT mice than in CMAH^−/−^ mice, respectively, although the differences did not reach statistical significance. In contrast, the liver:skeletal muscle transduction ratio for AAV6 in WT mice was 4.6-fold lower than in CMAH^−/−^ mice. Astonishingly, the heart:skeletal muscle and liver:skeletal muscle ratios of transduction for AAV5 were 57.3-fold (*p* < 0.001) and 14-fold (*p* < 0.01) higher in WT mice when compared to CMAH^−/−^ mice. As expected, none of the organ transduction ratios were statistically significantly different between WT and CMAH^−/−^ mice administered AAV9, although we observed a non-significant 4.3-fold higher heart:liver transduction ratio in CMAH^−/−^ mice compared to WT mice. Together, these results suggest that the distinct glycosylation pattern of CMAH^−/−^ mice compared to WT mice affects the tropism of *N*-linked SIA-binding AAV vectors.

## Discussion

In this study, we assessed the tropism of *N*-linked SIA-binding AAV1, 5 and 6 in CMAH^−/−^ mice that, like humans, lack the SIA Neu5Gc. Immunostaining of WT and CMAH^−/−^ mice tissue for Neu5Gc confirmed the overall lack of Neu5Gc in CMAH^−/−^ mice compared to WT mice in the major organs (heart, liver, skeletal muscle, brain, kidney, and lung). Lectin Blots of protein extracts and staining of tissue sections with the lectins MAL II or SNA showed that the abundance and staining pattern of α2–3 and α2–6 linked SIA-containing glycoproteins is, for the most part, comparable between CMAH^−/−^ and WT mice. The assessment of the transduction profile for AAV1, 5 and 6 in different organs of CMAH^−/−^ mice demonstrated that AAV1, 5 and 6 appear to prefer glycans with Neu5Gc vs. Neu5Ac to transduce heart tissue. In contrast, transduction of skeletal muscle by AAV5 was dramatically higher in CMAH^−/−^ mice when compared to WT mice. Our results demonstrate that the absence or presence of CMAH (and hence Neu5Gc; Figure 1) profoundly affects the tropism of AAVs that use *N*-linked SIA as their primary receptor. We chose luciferase as a reporter gene because it allows accurate measurement of transgene expression levels in various tissues. However, this reporter also has limitations. Chief among them is that it is challenging to determine the percentage and nature of cells that are transduced in any given tissue, which would yield information that cannot be gained from measurement of the overall transgene expression (42, 43). Future experiments, which are beyond the scope of the current study, with a different reporter protein (e.g., GFP) will be required to acquire such data.

The analysis of the mechanism(s) dictating the different transduction profiles in CMAH^−/−^ compared to WT mice upon systemic injection of *N*-linked SIA-binding AAV1, 5 and 6 is beyond the scope of this study. However, since α2–3 and α2–6 *N-*linked SIA have been previously shown to serve as receptors for AAV1, 5 and 6 (13, 14), our data suggest that the type of linkage (α2–3 vs. α2–6) between the sugars that form glycoproteins is not the only determinant that influences binding of AAVs to SIA, but that particular modifications on the sugars, such as the hydroxyl group that is present on Neu5Gc but missing on Neu5Ac, may also influence these interactions. Future studies are required to start unraveling the mechanism(s) by which the structural differences between Neu5Ac and Neu5Gc SIA alter AAV tropism *in vivo*. For instance, biodistribution studies of vector genomes of *N*-linked SIA-binding AAV serotypes in WT and CMAH^−/−^ mice will provide valuable data on whether the altered primary receptor affects AAV uptake, or if intracellular events are responsible for the observed differences in transduction efficiency. For instance, it will be interesting to analyze the influence of other host entry factors [e.g., AAVR or GRP108] (16, 44–46) on transduction in the two mice strains. Finally, given the wide range of doses that are currently used in clinical trials and evidence that vector dose can influence AAV tropism (47, 48), it will be important to assess whether the differences that we observed in this study may be dose-dependent.

AAV serotypes display complex interactions between the viral capsid and their receptors on host cells, which likely influences AAV tropism, especially upon systemic delivery to the bloodstream. Currently, the most commonly used animal models for the study of AAV candidates for gene therapy are mice, rats, pigs, dogs and non-human primates such as rhesus macaques, among others (1, 49). However, comparative studies of AAV receptors across species have shown profound differences. Importantly, several studies have consistently demonstrated how variable the sialylation pattern is among rodents, primates and humans (3, 4, 20). Since differences in the sialylation pattern of tissues can be associated to genetic divergence among species on genes related to the SIA biology (21), we propose that these genetic differences should be considered when selecting the animal model for each particular study. For instance, the influence of the absence of CMAH in humans on AAV tropism *in vivo*, as demonstrated in this study, emphasizes the importance of considering the nature of SIA when choosing animal models for preclinical studies.

Several species of mammals, including ferrets and marmosets have been documented to also lack Neu5Gc due to loss-of-function mutations that affect expression of CMAH (30–33). Ferrets have been used as models for the investigation of the SIA-binding influenza virus for decades (50) and have also been shown to be susceptible to AAV1, 2 and 9 transduction in the lung and trachea, eye, and nasal epithelia, respectively (51–54). Similarly, marmosets have also been used in AAV research. However, most of the research in marmosets has been performed by local injection, making the evaluation of tissue/cell tropism difficult, if not impossible (55–58). In one study, however, AAV1 and AAV9 were injected intraperitoneally into neonatal marmosets (59). This study showed transduction of several organs by both serotypes (skeletal muscle, liver, heart, kidney, ovaries, and brain with AAV1, and heart, liver, diaphragm, kidney, ovaries, and brain with AAV9), although the relative transduction efficiency varied. For instance AAV1 robustly transduced skeletal muscle, whereas AAV9 showed strong transduction of cardiomyocytes (59).

Given the dramatic differences in AAV tropism between WT and CMAH^−/−^ mice, in our view, to establish the most representative animal model for human tropism, it will likely be necessary to perform marker gene studies in humans with at least the serotypes currently used in the clinic and clinical trials (60). Only this type of studies will answer the question whether macaques (or any other species, for this matter) are the best model to predict the tropism of AAVs in humans. Since, like humans, New World monkeys such as the marmoset also lack CMAH, could they possibly be the better model for AAV tropism in humans?

## Data Availability Statement

The original contributions presented in the study are included in the article/Supplementary Material, further inquiries can be directed to the corresponding author/s.

## Ethics Statement

The animal study was reviewed and approved by the Institutional Animal Care and Use Committee (IACUC) of Icahn School of Medicine at Mount Sinai.

## Author Contributions

EL-G, AO, and TW contributed to conception and design of the study or its revision (SS). EL-G, AO, and AWa executed the experiments. EL-G, AO, and AWe performed the statistical analysis. EL-G wrote the first draft of the manuscript. AO and TW wrote sections of the manuscript. All authors contributed to manuscript revision, read, and approved the submitted version.

## Funding

This work was supported by NHLBI grant HL131404 (TW), R01HL148786 (SS), and R01HL140469 to (SS).

## Conflict of Interest

The authors declare that the research was conducted in the absence of any commercial or financial relationships that could be construed as a potential conflict of interest.

## Publisher's Note

All claims expressed in this article are solely those of the authors and do not necessarily represent those of their affiliated organizations, or those of the publisher, the editors and the reviewers. Any product that may be evaluated in this article, or claim that may be made by its manufacturer, is not guaranteed or endorsed by the publisher.

## Supplementary Material

Supplementary Figure 1 Neu5Gc staining of tissue sections of wild-type C57BL/6J (WT) or CMAH knockout (CMAH^−/−^) mice. Neu5Gc staining (blue) was performed on 6 μm paraffin-embedded heart, liver, skeletal muscle, brain, kidney or lung sections from male **(A)** and female **(B)** WT and CMAH^−/−^ mice with chicken anti-Neu5Gc antibody (cat. no.: 146903, BioLegend) or chicken IgY Isotype control (cat. no.: 402101, BioLegend) both at 1 ug/mL. Biotinylated goat anti-chicken IgY antibody (cat. no.: BA-9010, Vector Laboratories) was used as a secondary antibody. Nuclei were counterstained with VECTOR Nuclear Fast Red (red). Representative images are shown for each organ [*n* = 2 mice per group; magnification 10× (left panels) or 20× (right panels)]. Scale bars 100 μm (left panels) or 50 μm (right panels) for each organ.

Supplementary Figure 2 Lectin blotting analysis of sialic acid (SIA)-containing glycoproteins in mouse tissue protein extracts. Lectin staining was performed to visualize the SIA-containing glycoprotein staining profile in male heart, liver, skeletal muscle, brain, kidney, and lung protein extracts resolved on a 10% SDS-polyacrylamide gel. Maackia Amurensis Lectin (MAL) II was used to bind SIA in an α2–3 linkage (20 μg/ml) **(A)** and Sambucus Nigra Lectin (SNA) to bind SIA in an α2–6 linkage and to a lesser extent SIA in an α2–3 linkage (10 μg/ml) **(B)**. 15, 20, or 8 μg of protein extract from heart; liver, skeletal muscle and brain; or kidney and lung of wild-type C57BL/6J (WT) or CMAH knockout (KO) mice were loaded per lane, respectively. Precision Plus Protein Dual Color protein standard (Bio-Rad, USA) was used as a marker for molecular weight.

Supplementary Figure 3 Sialic acid (SIA)-containing glycoprotein staining of tissue sections of wild-type C57BL/6J (WT) or CMAH knockout (CMAH^−/−^) mice. Lectin staining (blue) was performed on 6 μm paraffin-embedded heart, liver, skeletal muscle, brain, kidney or lung sections from male **(A)** and female **(B)** WT and CMAH^−/−^ mice with Maackia Amurensis Lectin (MAL) II to bind SIA in an α2–3 linkage or Sambucus Nigra Lectin (SNA) to bind SIA in an α2–6 linkage and to a lesser extent SIA in an α2–3 linkage (10 μg/ml). Nuclei were counterstained with VECTOR Nuclear Fast Red (red). Representative images are shown for each organ [*n* = 1 mice per group; magnification 5× (left panels) or 20× (right panels)]. Scale bars 250 μm (left panels) or 50 μm (right panels) for each organ.
