# Supplementary figures and images for: Hydroxylation of N-acetylneuraminic Acid Influences the in vivo Tropism of N-linked Sialic Acid-Binding Adeno-Associated Viruses AAV1, AAV5, and AAV6
Source: Front Med (Lausanne). 2021 Dec 21;8:732095. doi: 10.3389/fmed.2021.732095 (PMC8757481; doi:10.3389/fmed.2021.732095)

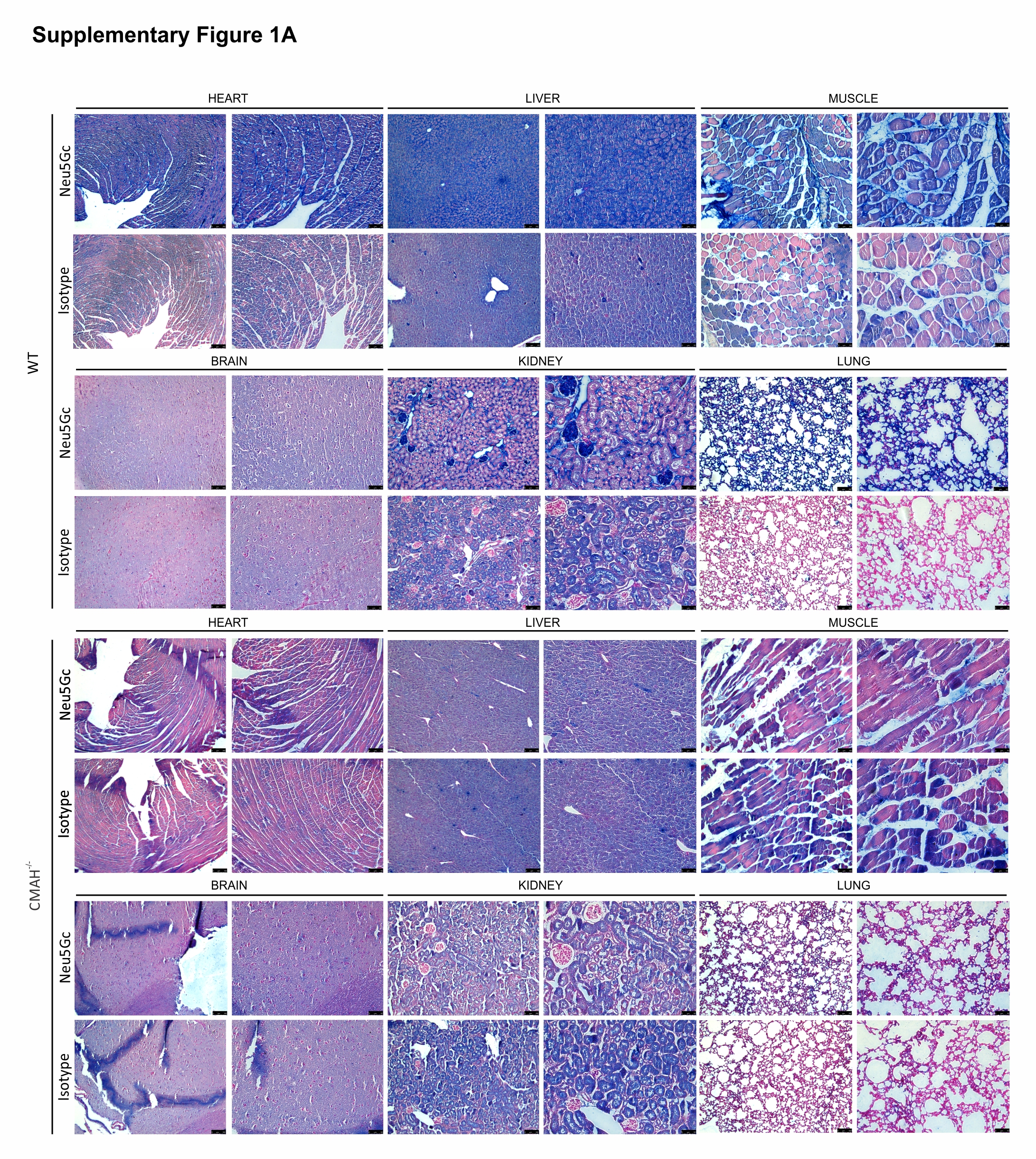

Supplement: Supplementary Figure 1 — Neu5Gc staining of tissue sections of wild-type C57BL/6J (WT) or CMAH knockout (CMAH−/−) mice. Neu5Gc staining (blue) was performed on 6 μm paraffin-embedded heart, liver, skeletal muscle, brain, kidney or lung sections from male (A) and female (B) WT and CMAH−/− mice with chicken anti-Neu5Gc antibody (cat. no.: 146903, BioLegend) or chicken IgY Isotype control (cat. no.: 402101, BioLegend) both at 1 ug/mL. Biotinylated goat anti-chicken IgY antibody (cat. no.: BA-9010, Vector Laboratories) was used as a secondary antibody. Nuclei were counterstained with VECTOR Nuclear Fast Red (red). Representative images are shown for each organ [n = 2 mice per group; magnification 10× (left panels) or 20× (right panels)]. Scale bars 100 μm (left panels) or 50 μm (right panels) for each organ. [file Image_1.jpg]

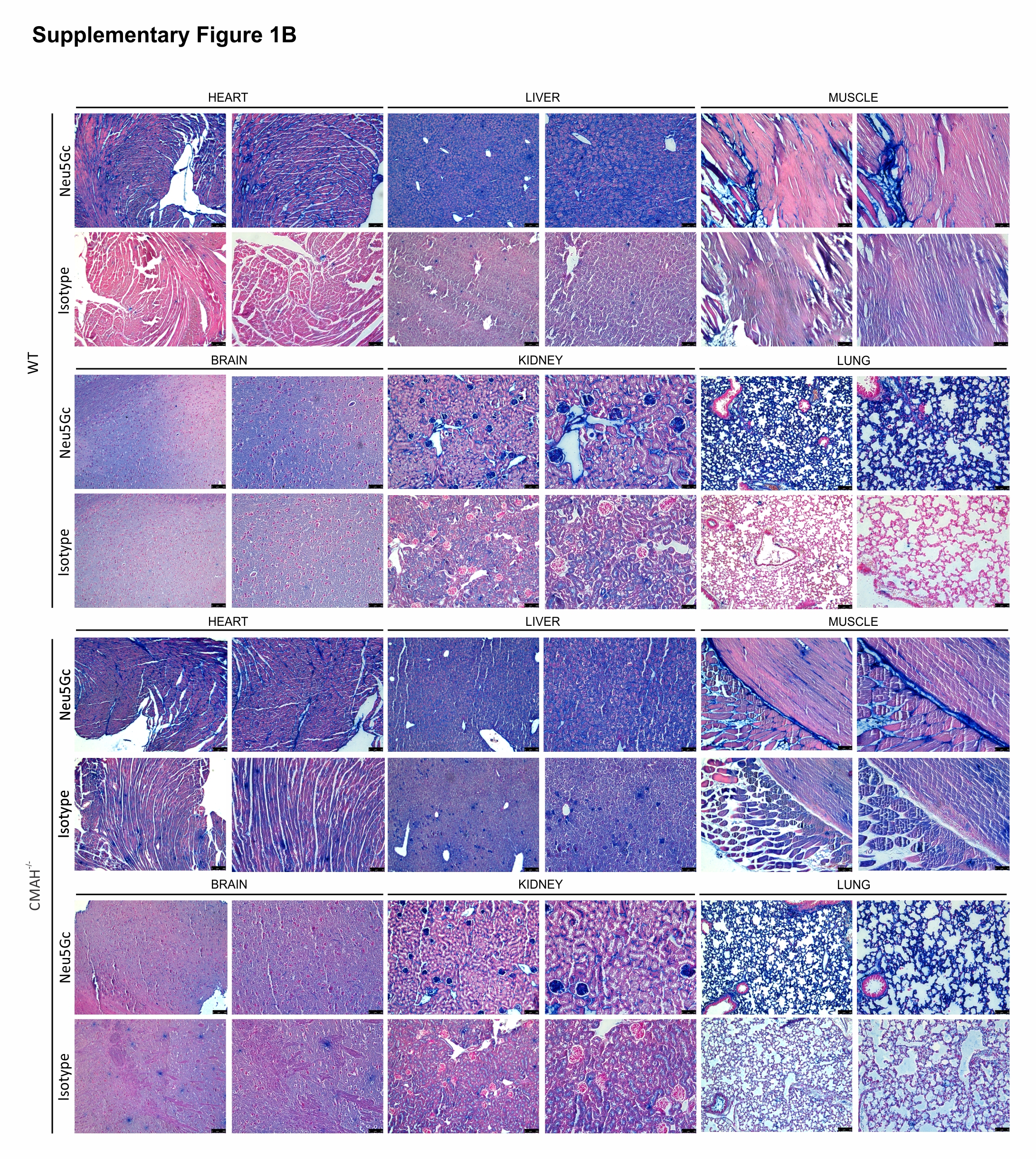

Supplement: Supplementary file 2 [file Image_2.jpg]

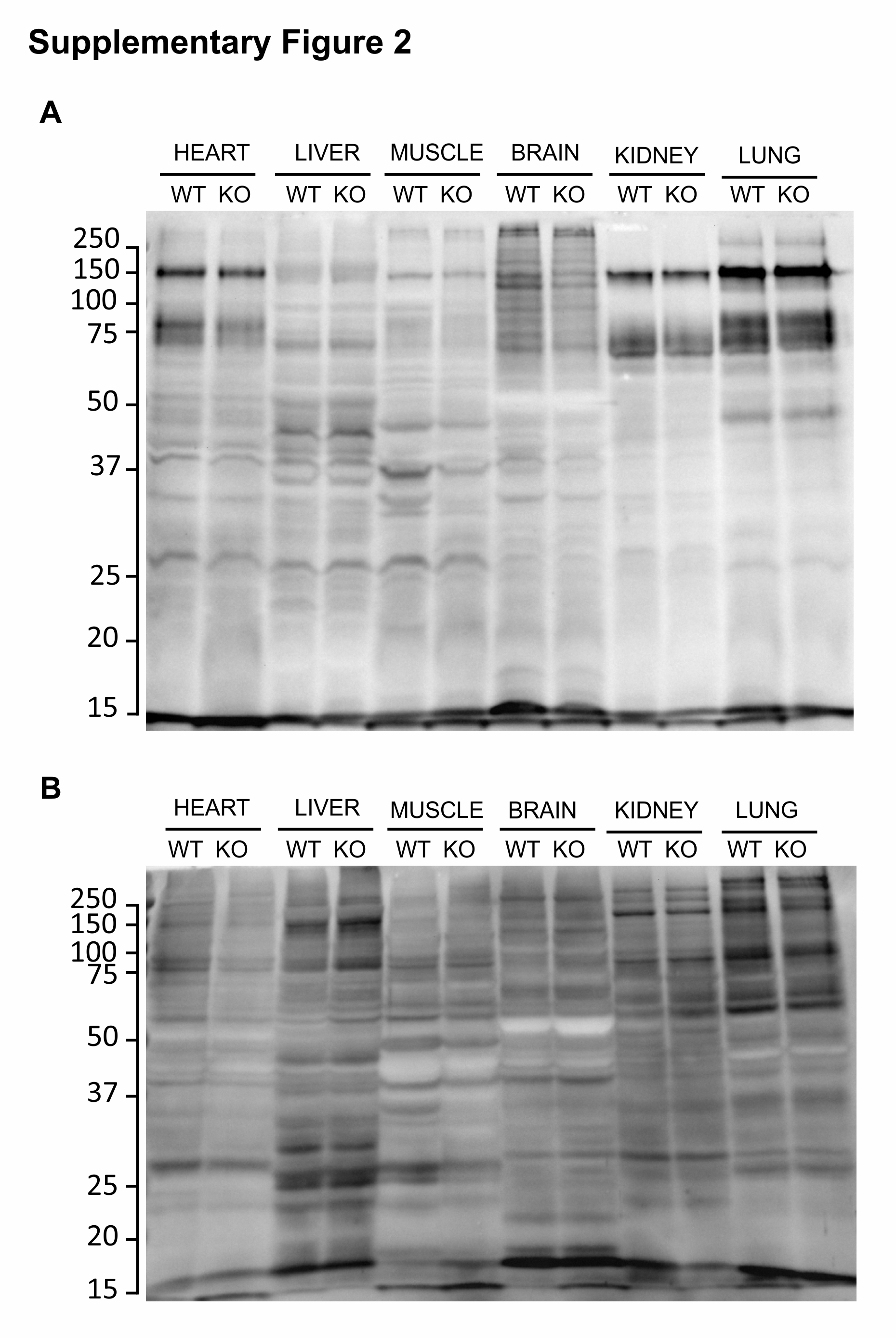

Supplement: Supplementary Figure 2 — Lectin blotting analysis of sialic acid (SIA)-containing glycoproteins in mouse tissue protein extracts. Lectin staining was performed to visualize the SIA-containing glycoprotein staining profile in male heart, liver, skeletal muscle, brain, kidney, and lung protein extracts resolved on a 10% SDS-polyacrylamide gel. Maackia Amurensis Lectin (MAL) II was used to bind SIA in an α2–3 linkage (20 μg/ml) (A) and Sambucus Nigra Lectin (SNA) to bind SIA in an α2–6 linkage and to a lesser extent SIA in an α2–3 linkage (10 μg/ml) (B). 15, 20, or 8 μg of protein extract from heart; liver, skeletal muscle and brain; or kidney and lung of wild-type C57BL/6J (WT) or CMAH knockout (KO) mice were loaded per lane, respectively. Precision Plus Protein Dual Color protein standard (Bio-Rad, USA) was used as a marker for molecular weight. [file Image_3.jpg]

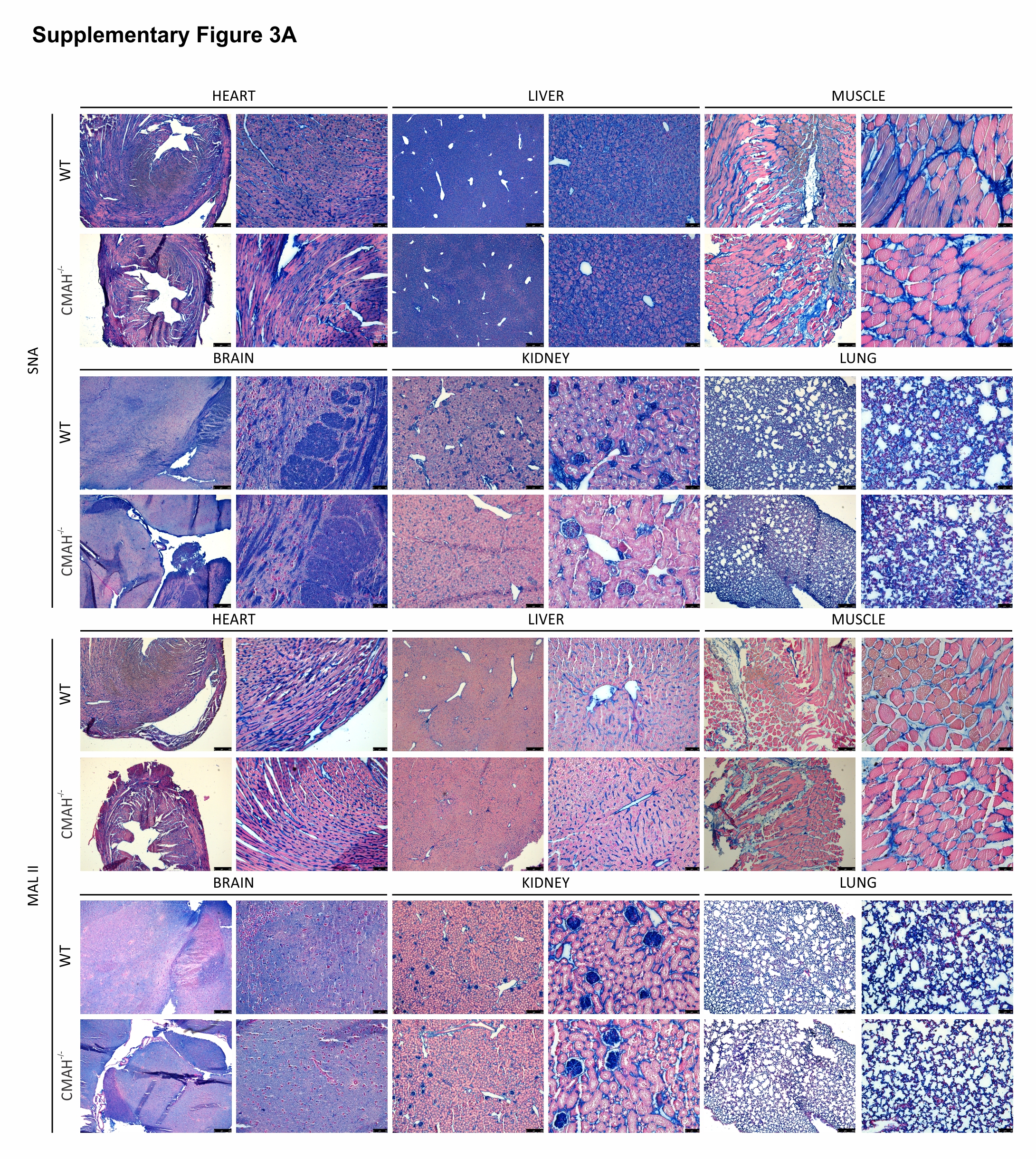

Supplement: Supplementary Figure 3 — Sialic acid (SIA)-containing glycoprotein staining of tissue sections of wild-type C57BL/6J (WT) or CMAH knockout (CMAH−/−) mice. Lectin staining (blue) was performed on 6 μm paraffin-embedded heart, liver, skeletal muscle, brain, kidney or lung sections from male (A) and female (B) WT and CMAH−/− mice with Maackia Amurensis Lectin (MAL) II to bind SIA in an α2–3 linkage or Sambucus Nigra Lectin (SNA) to bind SIA in an α2–6 linkage and to a lesser extent SIA in an α2–3 linkage (10 μg/ml). Nuclei were counterstained with VECTOR Nuclear Fast Red (red). Representative images are shown for each organ [n = 1 mice per group; magnification 5× (left panels) or 20× (right panels)]. Scale bars 250 μm (left panels) or 50 μm (right panels) for each organ. [file Image_4.jpg]

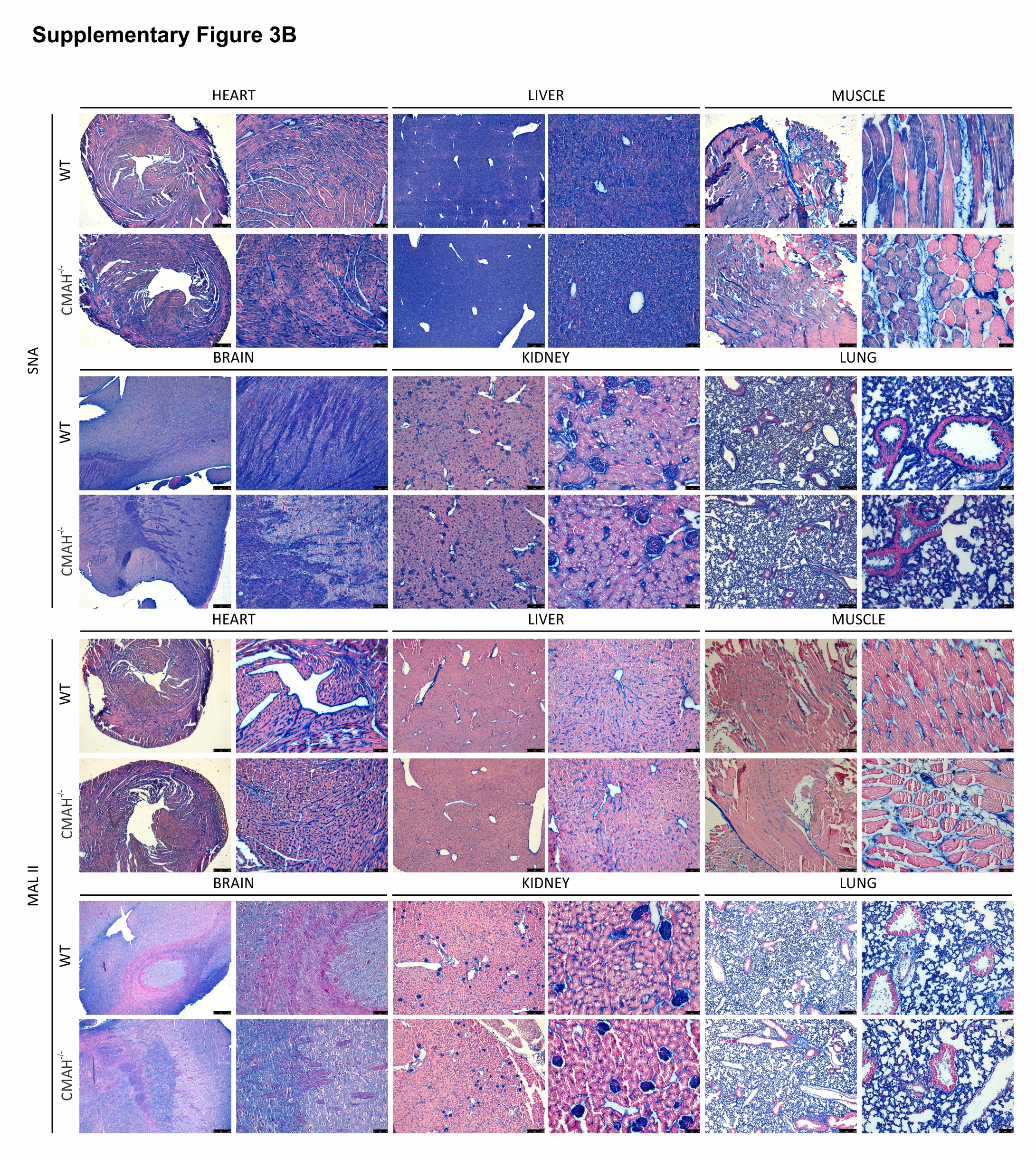

Supplement: Supplementary file 5 [file Image_5.jpg]
